# Predicting Modulation in Corticomotor Excitability and in Transcallosal Inhibition in Response to Anodal Transcranial Direct Current Stimulation

**DOI:** 10.3389/fnhum.2016.00049

**Published:** 2016-02-15

**Authors:** Travis W. Davidson, Miodrag Bolic, François Tremblay

**Affiliations:** ^1^School of Human Kinetics, University of OttawaOttawa, ON, Canada; ^2^Faculty of Engineering, School of Electrical Engineering and Computer Science, University of OttawaOttawa, ON, Canada; ^3^Faculty of Health Sciences, School of Rehabilitation Sciences, University of OttawaOttawa, ON, Canada; ^4^Clinical Neuroscience Lab, Bruyère Research InstituteOttawa, ON, Canada

**Keywords:** non-invasive brain stimulation, motor evoked potential, motor cortex, transcranial magnetic stimulation, transcranial direct current stimulation (tDCS)

## Abstract

**Introduction**: Responses to neuromodulatory protocols based either on transcranial direct current stimulation (tDCS) or transcranial magnetic stimulation (TMS) are known to be highly variable between individuals. In this study, we examined whether variability of responses to anodal tDCS (a-tDCS) could be predicted from individual differences in the ability to recruit early or late indirect waves (I-waves), as reflected in latency differences of motor evoked potentials (MEPs) evoked by TMS of different coil orientation.

**Methods**: Participants (*n* = 20) first underwent TMS to measure latency of MEPs elicited at different coil orientations (i.e., PA, posterior-anterior; AP, anterior-posterior; LM, latero-medial). Then, participants underwent a-tDCS (20 min @ 2 mA) targeting the primary motor cortex of the contralateral preferred hand (right, *n* = 18). Individual responses to a-tDCS were determined by monitoring changes in MEP amplitude at rest and in the duration of the contralateral silent period (cSP) and ipsilateral silent period (iSP) during contraction; the latter providing an index of the latency and duration of transcallosal inhibition (LTI and DTI).

**Results**: Consistent with previous reports, individual responses to a-tDCS were highly variable when expressed in terms of changes in MEP amplitude or in cSP duration with ~50% of the participants showing either little or no modulation. In contrast, individual variations in measures of transcallosal inhibition were less variable, allowing detection of significant after-effects. The reduced LTI and prolonged DTI observed post-tDCS were indicative of an enhanced excitability of the transcallosal pathway in the stimulated hemisphere. In terms of predictions, AP-LM latency differences proved to be good predictors of responses to a-tDCS when considering MEP modulation.

**Conclusion**: The present results corroborate the predictive value of latency differences derived from TMS to determine who is likely to express “canonical” responses to a-tDCS in terms of MEP modulation. The results also provide novel suggestive evidencethat a-tDCS can modulate the excitability of the transcallosal pathway of the stimulated hemisphere.

## Introduction

Transcranial direct current stimulation (tDCS) has gained much credentials in recent years as a neuromodulatory intervention to induce lasting changes in cortical excitability. By passing a very weak current (e.g., 1–2 mA) through surface electrodes applied on the scalp, one can attempt to modulate the excitability of the underlying cortical region presumably by acting of resting membrane potential (Dayan et al., 2013). Anodal tDCS (a-tDCS) tends to increase the spontaneous firing rate and the excitability of cortical neurons by depolarizing the membrane, whereas cathodal stimulation leads to hyperpolarization of the neuronal membrane and thus tends to decrease excitability (Stagg and Nitsche, 2011; Dayan et al., 2013). One of the major drawbacks in this simplistic model is that stimulation-induced behavioral and physiological effects tend to vary substantially from one individual to another (for a review, see Horvath et al., 2014). In the case of tDCS, this variability has been highlighted in two recent reports (López-Alonso et al., 2014; Wiethoff et al., 2014) where after-effects on cortical excitability were monitored using motor evoked potentials (MEPs) derived from transcranial magnetic stimulation (TMS). In both reports, only ~half of the participants responded in the canonical manner to tDCS applications and exhibited the expected modulation in terms of either MEP facilitation (anodal) or depression (cathodal). As stressed by the authors of these reports, such a large inter-individual variability has implications not only for studies examining behavioral and physiological effects of neuromodulatory protocols but also for studies interested in the therapeutic potential of tDCS in clinical populations.

While differences in experimental protocols (e.g., electrode types, stimulator settings) might account for some of the variability, individual differences in anatomical factors such as the thicknesses of the cerebrospinal and the skull can also contribute significantly, as they are known to have a direct influence on the current flow that reaches the cortex (Datta et al., 2009, 2012; Laakso et al., 2015). Another potential and important source of variability highlighted in a recent study by Hamada et al. (2013) when examining MEP modulation in response to theta burst stimulation (TBS) was individual susceptibility to activate certain population of cortical interneurons by TMS. This conclusion was based on observations regarding differences in MEP latency evoked by monophasic TMS pulses of different coil orientations to assess how easily direct waves (D-wave) or indirect waves (I-waves) can be recruited in a given individual. Indeed, previous *in vivo* recordings in humans have shown that, depending on the coil orientation and intensity, monophasic TMS pulses can evoke different combinations of descending waves, which reflect different cortical generators (Di Lazzaro et al., 2004). For instance, high intensity latero-medial (LM) induced currents tend to evoke D-wave and early I-waves (Sakai et al., 1997; Di Lazzaro et al., 2004). The D-waves are termed “direct” because they are thought to originate from direct activation of axons of layer V pyramidal tract neuron (Di Lazzaro et al., 2004). Correspondingly, I-waves are considered “indirect” because they are thought to result from indirect, transynaptic activation of pyramidal tract neurons (Di Lazzaro et al., 2012). With conventional posterior-anterior (PA) currents, early I-waves (I1-I2) can be easily elicited even with low intensity stimulation, probably reflecting activation of low-threshold cortical elements in layers II and III making mono and oligo-synaptic contacts with pyramidal tract neurons (Di Lazzaro et al., 2012). In contrast, anterior-posterior (AP) currents tend to evoke only later I-waves (I3, I4) owing to their presumed polysynaptic origin in link with activation of a network composed of cortical elements in upper layers (II and III) and local interneurons acting on pyramidal tract neurons through reciprocal excitatory and inhibitory connections (Di Lazzaro et al., 2012). Following this rationale, Hamada et al. (2013) used differences in MEP latency evoked at the AP and LM orientation (i.e., AP-LM differences) as a surrogate measure of individual susceptibility to recruit early or late I-waves in response to TMS and by inference, activate different population of cortical interneurons. With this approach, Hamada et al. (2013) were able to predict with great accuracy who was likely to express either lasting facilitation or lasting depression in response to neuromodulatory TBS applications based on how readily early or late I-waves could be recruited, as reflected in AP-LM latency differences. In a subsequent study, the same group (Wiethoff et al., 2014) used a similar approach to examine inter-individual variability in response to tDCS. As noted before, MEP modulation in response to either anodal or cathodal stimulation was quite variable but, interestingly, here again AP-LM latency differences proved to be a good predictor of whom was likely to exhibit “canonical” responses to tDCS. The prediction was particularly compelling for individuals showing small AP-LM differences and thus, D-wave or early I-waves recruitment, in whom MEP facilitation was large and consistent after anodal stimulation.

Thus, one way to tackle inter-individual variability in response to neuromodulatory protocols may consist in identifying in a given individual and prior to application which populations of cortical interneurons are likely to be modulated. In the present report, we sought to further investigate this question along the path opened by Wiethoff et al. (2014) to examine whether variability of responses to a-tDCS could be predicted by individual susceptibility to recruit early or late I-waves, as reflected in MEP latency differences. To this end, we monitored changes in corticomotor excitability before and after tDCS using MEP at rest since they provide the most reliable outcomes of neuromodulatory protocols (Horvath et al., 2015). In addition to MEPs, we also monitored changes in the contralateral and ipsilateral silent period (cSP and iSP, respectively) during active contractions. The cSP provides another index of corticomotor excitability reflecting modulation of central inhibition via γ-amino butyric acid (GABA) B receptors (Ziemann et al., 2015). The iSP was recorded to assess changes in transcallosal inhibition (Meyer et al., 1995) from the stimulated hemisphere towards the non-stimulated hemisphere.

## Materials and Methods

### Participants

Twenty healthy adults (mean age 24.3 ± 7.3 years, 15 men) were recruited from the local community, most being university students in the Ottawa area. Before testing, all participants were screened with a safety questionnaire (adapted from Keel et al., 2001) to ensure that there were no contra-indications to TMS. The majority (18/20) were right handed based on the Edinburg Handedness Inventory. Prior to participation, written informed consent was obtained from all participants in accordance with the *Declaration of Helsinki* and the study procedures were approved by the local institutional Research Ethics Board (Bruyère Research Institute, Ottawa, ON, Canada). All assessments were performed in a controlled laboratory environment and participants received a small honorarium for their participation.

#### Experimental Paradigm

**Figure 1:**
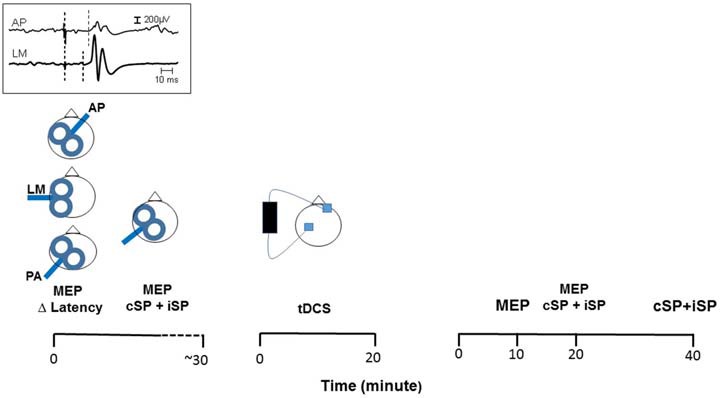
**Schematic representation of the experimental paradigm.** Participants (*n* = 20) first underwent transcranial magnetic stimulation (TMS) to determine latency of motor evoked potentials (MEPs) using three different coil orientations: anterior-posterior (AP), latero-medial (LM) and posterior-anterior (PA). An example of differences in MEP latency evoked in AP and LM orientation is shown in the inset. Then, participants underwent TMS again to determine baseline value for the MEP amplitude at rest and the contralateral and ipsilateral silent period (cSP and iSP) during contraction. Then, anodal transcranial direct current stimulation (a-tDCS) was applied (20 min, 2 mA) with the anode placed on the motor cortex contralateral to the preferred hand (right, *n* = 18) and the cathode placed on the opposite supra-orbital region. Finally, to assess the after-effect of tDCS, changes in the MEPs at rest were measured at 10 and 20 min post-tDCS. At the 20th min, the cSP and iSP were also measured and then again at 40th min post.

The experimental paradigm is illustrated in Figure 1. Participants first underwent TMS testing to measure MEP latency with three different coil orientations, i.e., PA, LM and AP. Then, baseline measures of corticomotor excitability were performed to determine MEP characteristics at rest using the conventional PA coil orientation. The cSP and iSP were also measured concurrently during active contractions. Following baseline measurements, participants underwent a-tDCS for 20 min. Then, MEPs were measured again at 10 min (T10) and 20 min (T20) post-application. At T20, the cSP and iSP were also measured and then again at 40 min post-application (T40). The delayed testing till the 20th min for the cSP and iSP was introduced to avoid possible interferences with measurements of MEPs at rest associated with active contractions (i.e., post-contraction changes in excitability, see Goldsworthy et al., 2014).

#### Pinch Strength Measurements During Maximum Voluntary Contraction (MVC)

Prior to neurophysiological testing, participants were tested with a mechanical pinch gauge (Model, 12-0201, Fabrication Enterprises Inc., Irvington, NY, USA) during maximal efforts to measure strength and obtain an index of muscle activation in the first dorsal interosseous (FDI) muscle. For pinch strength, participants were instructed to press as hard as they could against the dynamometer using a lateral “key” pinch with the thumb and index fingers. Participants were instructed to synchronize their effort with an auditory tone lasting 3 s generated by the computer. Three trials were recorded for each hand with at least 30 s rest between contractions. The order of testing with the right and left hand alternated between participants.

#### General Procedure for TMS and MEP Recordings

TMS was administered with participants comfortably seated in a recording chair. Participants were fitted with a Waveguard TMS compatible cap (ANT North America Inc., WI, USA) to allow localization and to ensure consistent coil positioning. A U-shaped neck cushion was also used to restrain head movements and prevent neck fatigue. Magnetic stimulation was delivered via Magstim 200 monophasic stimulator (Magstim Co., Dyfed, UK) connected to a figure-eight coil (90 mm outer loop diameter). MEPs were recorded using small auto-adhesive surface electrodes (Ag/AgCl, Kendall Medi-Trace^TM^ 130) placed in a belly-tendon montage on the FDI muscles of each hand. Electromyographic (EMG) signals were amplified and filtered with a time constant of 0.03 s and a low-pass filter of 1 kHz (AB-621G Bioelectric amplifier, Nihon-Kohden Corp., CA, USA). Signals were digitized at rate of 2 kHz (PCI-6023E, BNC-2090, National Instruments Corp) and further relayed to a laboratory computer running custom software to control acquisition and saved for offline analysis.

Each TMS testing session proceeded with the same sequence for all participants. First, the motor hot spot for the FDI was determined on each hemisphere, starting with the hemisphere contralateral to the preferred hand. With the coil held ~45° in the mid-sagittal plane, the approximate location of the hand motor area was explored in 1 cm steps until reliable MEPs could be evoked in the FDI. This site was then marked with a circular sticker to ensure consistent coil positioning throughout the testing session. After determination of the “hotspot”, the resting and and active motor thresholds (rMT and aMT) were determined using the Motor Threshold Assessment Tool software (MTAT 2.0^1^). The software allows for fast estimation of motor threshold through the maximum-likelihood strategy based on the PEST (Parameter Estimation by Sequential Testing) algorithm (Mishory et al., 2004). For the rMT, participants were instructed to relax while EMG was monitored on an oscilloscope to ensure that no unwanted contraction was present. For the aMT, TMS pulses were delivered while the FDI was lightly activated by asking participants to press against the pinch dynamometer with a low force corresponding to ~10% of their maximum. For the sequential testing algorithm, the minimal acceptable MEP amplitude was set at 50 μV for the rMT and at 200 μV for the aMT. Once completed for one hemisphere, the same procedure for determination of the hotspot, rMT and aMT was repeated on the opposite hemisphere.

#### Assessment of MEP Latency with Different Coil Orientation

Following Hamada et al. (2013), the relative efficiency in recruiting early vs. late I-waves in a given individual was estimated by eliciting MEPs at three different coil orientations. As described earlier, current induced with the PA direction tends to recruit early I-waves, while current induced with the AP direction tends to recruit later I-waves (Ni et al., 2011). In the LM direction, strong TMS pulses produced MEPs with a latency close to those elicited by transcranial electrical stimulation and thus, are thought to reflect D-waves recruitment (Di Lazzaro et al., 2004). For the PA current, the coil was held tangentially to the scalp in the usual orientation, i.e., with the handle pointing backward and the coil oriented ~45° in the mid-sagittal plane. For the LM orientation, the coil was applied on the hot spot with the handle pointing downward in line with the inter-aural line. For the AP current, the coil was simply rotated ~180° form the usual PA orientation with the handle pointing anteriorly. At each orientation, 10 MEPs were elicited by stimulating the hemisphere contralateral to the preferred hand (right, *n* = 18) and while the FDI was actively contracting using the pinching task described above (~10% MVC). The stimulation intensities at each orientation were based on the aMT previously determined (see preceding section). For the PA orientation the intensity was set at 110% of aMT, whereas for the LM and AP orientation the intensity was set at 140% aMT. The order of testing with each coil orientation was counterbalanced across participants. Examples of MEP latency evoked at the different coil orientations can be seen in Figure 1.

#### Baseline MEP Amplitude and cSP/iSP Assessment

After testing with the different coil orientations, baseline MEP amplitude at rest was determined by recordings 10 MEPs in response to single TMS pulses delivers at 120% rMT on the hemisphere (left, *n* = 18) contralateral to the preferred hand. Then, the cSP and iSP were assessed concurrently as described previously (Davidson and Tremblay, 2013a). Briefly, participants were instructed to press as hard as they could on a pinch dynamometer with their preferred hand (right, *n* = 18), while exerting a light constant pinching force (~25% of the maximal strength) with their opposite hand on a second dynamometer. Participants were trained to maintain the contractions for 3 s in synchronization with a tone (550 Hz) generated by the computer. In each trial, a supra-threshold (130% rMT) TMS pulse was delivered on the hemisphere ipsilateral to the maximally contracting hand at 2 s in the course of the 3 s trial. From this hemispheric stimulation, an iSP could be elicited in the ipsilateral hand (maximal contraction) while, at the same time, a cSP with the accompanying MEP could be recorded in the opposite hand (light contraction). Such procedure was repeated five times to get a sample of iSP and cSP recordings with an interval of at least 60 s between trials to prevent fatigue.

#### Anodal tDCS (a-tDCS) Intervention

The a-tDCS intervention was performed using the typical montage to induce changes in corticomotor excitability (Nitsche and Paulus, 2000) with the anode (35 cm^2^) positioned over the FDI motor hotspot (left, *n* = 18) and the cathode (100 cm^2^) in the contralateral supra-orbital area. Prior to application, the electrodes were placed in sponges previously soaked with saline solution (0.9% sodium chloride, Baxter, Corp., Toronto, ON, Canada). The a-tDCS was produced using a SmartStim Model 200^2^ (NorDocs Technologies Inc., Sudbury, ON, Canada) and consisted of 2 mA current applied for 20 min with a 30 s ramp-up and ramp-down. Participants were asked to fill a brief questionnaire both during (5 min) and after the stimulation to monitor for possible side effects.

#### Post-tDCS Assessment

Once the stimulation completed, participants underwent TMS at T10 and T20 to assess change in resting corticomotor excitability by recording 10 MEPs @ 120% rMT. At T20, and once MEPs had been recorded, the cSP and iSP were assessed during active contractions. These measures were again repeated at T40. As indicated before, the joint assessment of the cSP and iSP was delayed until the 20th min to avoid possible confound with assessment of excitability at rest owing to post-contraction after-effects (Goldsworthy et al., 2014).

### Data Analysis

All the analyses were performed offline by the same investigator (TD). The analysis was carried out in three steps. First, MEPs trials recorded with the different coil orientation (i.e., PA, LM, AP) were superimposed in each participant to determine their onset latency using visual inspection. From this data set, the AP-LM and PA-LM latency differences were computed in each participant to estimate the relative ease of recruiting early and late I-waves, respectively. Second, MEPs recorded at baseline and at each interval post-tDCS were averaged to derive mean amplitude values for each participant. Finally, cSP and iSP trials were analyzed to measure duration and other parameters. For this analysis, a trial-by-trial approach was performed. For trials with the contralateral hand (light contraction), the cSP duration was determined as the time interval from the onset of the MEP to the return of at least 50% of the mean pre-stimulus background EMG activity. Note that the size of the facilitated MEP (peak-to-peak) in each trial was also measured but was not considered as an outcome in this study. For trials with the ipsilateral hand, the iSP onset and offset were determined to derive two measures of transcallosal inhibition. The iSP onset was determined as the time from the stimulus onset until the 1st sign of significant decline (i.e., ≥25%) in the mean rectified EMG activity from pre-stimulus level for at least a 5 ms duration (i.e., 10 consecutive sampling points at 2 kHz), whereas the iSP offset was determined as the 1st sign of sustained recovery (>5 ms in duration) in the background EMG activity. The latter time point is usually easy to determine, as the end of the myoelectric silence is generally followed by an abrupt return of EMG activity in the recovery period (Davidson and Tremblay, 2013a,b). From these two time points, we used the iSP onset as an index of the latency of transcallosal inhibition (LTI) and the difference in ms between the offset and onset as an index of the duration of transcallosal inhibition (DTI).

### Statistical Analysis

To examine the variability of inter-individual responses, variations measured at each time point post-tDCS were averaged for all dependent variables (i.e., MEP amplitude, cSP duration, LTI and DTI) to get a grand average. The grand average was then expressed as a ratio relative to baseline for each participant. To assess the overall effect of a-tDCS, one-way repeated-measures analyses of variance (ANOVA) was performed on each dependent variable to detect main effect. Upon detection of main effect, the Dunnett’s multiple tests was performed to locate significant differences from baseline. MEP amplitude data were not normally distributed (Shapiro-Wilk test *p* < 0.05) and had to be log-transformed before entering the ANOVA. Finally, linear regression analyses were performed to examine the relationship between MEP latency differences and individual responses to a-tDCS expressed as ratios for all dependent variables. In addition, following the observations of Wiethoff et al. (2014), the predictive value of baseline MEP amplitude was also examined using regression analysis. The level of significance was set at *p* < 0.05 for all tests. All the analyses and graphical illustrations were performed using GraphPad Prism version 6.00 for Windows (GraphPad Software, La Jolla, CA, USA^3^).

## Results

### General Observations, Motor Threshold and MEP Latency

In general, both the TMS and tDCS were well tolerated. During the tDCS application, most participants reported tingling (60%) and itching 40%, but these effects were rated as mild to moderate on a 6-point scale. The average threshold for the rMT and aMT was respectively 37.5% (±6.0) and 32.1% (±2.5). As expected, the MEP onset latency measured with the LM orientation (mean 19.4 ± 1.5 ms) was, on average, 1.4 and 2.6 ms shorter than that measured with either the PA (20.8 ± 1.6 ms) or AP orientation (22.0 ± 1.5 ms).

### Effect of tDCS on MEP Modulation and cSP in the Contralateral Hand

**Figure 2:**
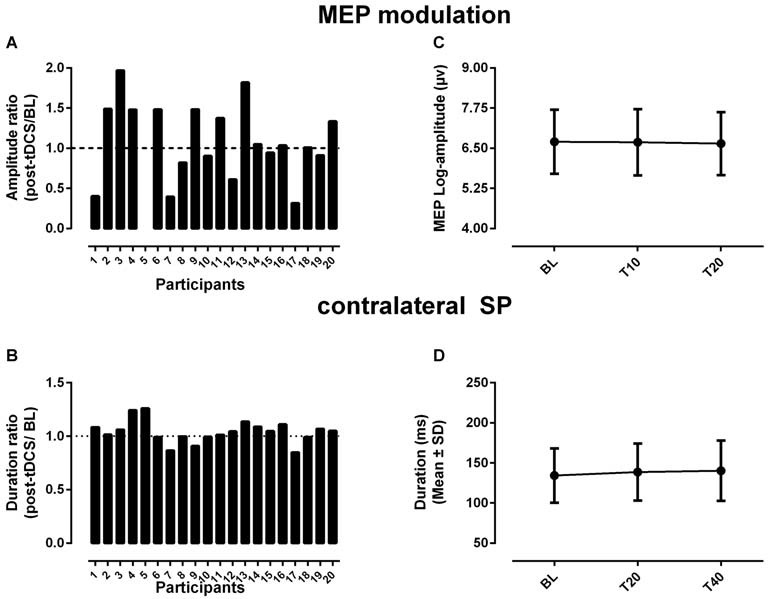
**Changes in MEP amplitude (A,C) and in the contralateral silent period (SP) duration (B,D) in response to a-tDCS.** In **(A,C)** changes are shown for each individual as a ratio of the grand average computed for all time points post-tDCS divided by baseline (i.e., post-tDCS/BL). The dotted lines in each graph indicate no change relative to baseline. Note that the missing data **(A)** refers to an outlier (participants #5) whose MEP data had to be removed from the analysis. In **(B,D)** the overall changes are shown as means and standard deviations computed across all participants and for each time point before (BL) and after a-tDCS. Note the relatively large variability observed between individuals for modulation in MEP amplitude **(A)** and in cSP duration **(C)**.

Individual responses to tDCS were quite variable when considering changes in MEP amplitude. This variability is evident by inspecting Figure 2A, where individual variations in MEP amplitude relative to baseline are shown for all participants. Note that MEP data from one participants (#5) had to be removed for her facilitation post-tDCS far exceeded the range observed in other participants (significant outlier, Grubb’s test, *Z* = 2.7, *p* < 0.05). Only ~40% of the participants (8/19) exhibited the expected pattern of MEP facilitation, while the remaining either showed a depression (*n* = 6) or minor changes in amplitude relative to baseline (*n* = 5). In view of this variability, no significant main effect (*F*_(2,18)_ = 0.13, *p* = 0.81) was detected when comparing MEP log-amplitude before and after a-tDCS (Figure 2C). With regards to changes in the cSP duration, the pattern of responses was characterized by a large proportion of “non-responders” (12/20) who showed little or no modulation relative to baseline, as can be seen in Figure 2B. Accordingly, the ANOVA failed to detect main effect (*F*_(2,19)_ = 2.2, *p* = 0.14) in the cSP duration in response to a-tDCS (Figure 2D).

### Effect of tDCS on Measures of Transcallosal Inhibition

**Figure 3:**
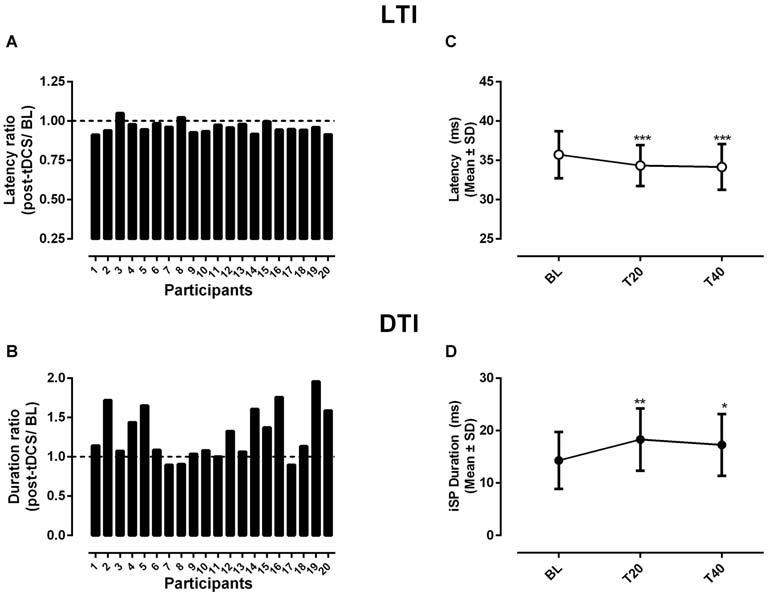
**Changes in the onset latency (A,C) and in the duration (B,D) of transcallosal inhibition (respectively, LTI and DTI) in response to a-tDCS.** In **(A,C)** changes are shown for each individual as a ratio of the grand average computed for all time points post-tDCS divided by baseline (i.e., post-tDCS/BL). The dotted lines in each graph indicate no change relative to baseline. In **(B,D)** the overall changes are shown as means and standard deviations computed across all participants and for each time point before (BL) and after tDCS. Note the significant changes in both LTI and DTI relative to baseline detected in post-test comparisons as indicated by the asterisks (*p < 0.05, **p < 0.01, ***p < 0.001).

As shown in Figure 3A, individual variations measured in LTI in response to a-tDCS showed less variability between participants than those seen for either MEPs or cSP. In fact, while the magnitude of changes tended to be small relative to baseline, the direction of change was highly consistent with 75% (15/20) of participants showing reduced LTI. The latter after-effect was confirmed by the ANOVA (*F*_(2,19)_ = 10.5, *p* = 0.001) and significant differences from baseline were detected for both T20 and T40 (Figure 3C). Although the direction of changes in DTI was less consistent than that seen for LTI, a majority of participants (13/20) exhibited an increase in duration, as shown in Figure 3B. The ANOVA confirmed that DTI was significantly changed post-tDCS (*F*_(2,19)_ = 8.97, *p* = 0.009) with significant differences from baseline being detected both at T20 and T40 (Figure 3D).

### Relationship Between MEP Latency Differences and Responses to tDCS

**Figure 4:**
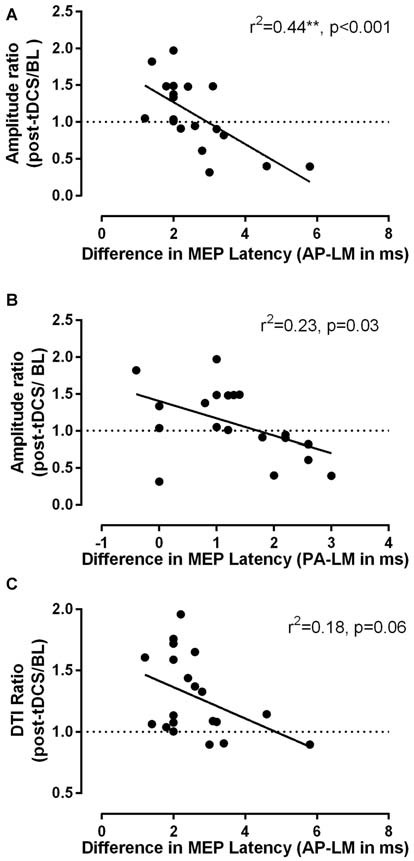
**Relationships between MEP latency differences measured with different TMS coil orientations (AP, LM, PA) and individual responses to a-tDCS.** In **(A,B)** the respective value of AP-LM and PA-LM latency differences in predicting variations in MEP amplitude is shown, while in **(C)**, the predictive value of AP-LM differences to explain variations in DTI is shown. Note that small differences in both AP-LM and PA-LM orientations were strongly predictive of the probability of exhibiting MEP facilitation (i.e., ratio > 1) in response to a-tDCS.

The results of the regression analysis to examine predictors of individual responses to a-tDCS are shown in Table 1. It can be seen that among the candidate variables only latency differences exhibited some degrees of association with variations observed in corticomotor excitability and in transcallosal inhibition following a-tDCS. AP-LM latency differences proved to be particularly strong predictors of MEP modulation, this factor accounting for >40% of the variance seen after tDCS. PA-LM latency differences were also good predictors of MEP modulation. Finally, AP-LM latency differences also tended to predict variations observed in DTI, but the relationship was only marginally significant (*p* = 0.06). In Figure 4, the association between latency differences and individual responses to tDCS can be further appreciated for both MEP (Figures 4A,B) and DTI (Figure 4C) variations.

**Table 1:** **Coefficient of determination (*r*^2^) computed from regression analysis for candidate predictors of individual variations in measures of corticomotor excitability and in transcallosal inhibition following anodal tDCS**.

	Measures of excitability derived from the contralateral hand		Measures of transcallosal inhibition derived from the ipsilateral hand	
Predictor	MEP (*n* = 19)	cSP	LTI	DTI
BL MEP^1^	0.13	0.12	<0.01	0.11
AP-LM LD	0.44**	0.13	<0.01	0.18
PA-LM LD	0.23*	0.01	0.02	0.01
AP-PA LD	0.09	0.11	0.02	0.17

Note that all dependent variables correspond to ratio values of the grand average post-tDCS/baseline. Also note that, except for baseline MEP (n = 19, 1 outlier), all correlations are based on 20 participants. Abbreviations: MEP, Motor evoked potential, cSP, contralateral silent period; LTI, latency of transcallosal inhibition; DTI, duration of transcallosal inhibition; BL, Baseline, AP-LM LD, Anterior-posterior-Latero-medial latency differences; PA-LM LD, Posterior-anterior-Latero-medial latency differences; AP-PA LD, Anterior-posterior-Posterior-anterior latency differences. Significant correlations are denoted in bold characters and asterisks (*p < 0.05), **p < 0.01.

## Discussion

In this study, we examined whether variability in response to a-tDCS in terms of changes in corticomotor excitability and in transcallosal inhibition could be predicted from individual susceptibility to activate early of late I-waves, as reflected in latency differences arising from TMS with different coil orientation. Consistent with other reports (López-Alonso et al., 2014; Wiethoff et al., 2014), individual responses in terms of changes in MEP amplitude were quite variable among participants leading to overall marginal after-effects. In comparison, changes in transcallosal inhibition appeared more consistent, leading to detection of significant changes in both LTI and DTI in the stimulated hemisphere post-tDCS. In addition, in agreement with Wiethoff et al. (2014), we showed that latency differences derived from TMS are indeed good predictors of individual changes in MEP amplitude observed after a-tDCS. In the following discussion, we will address the significance of these results for studies on neuromodulation and discuss the relationships observed between tDCS after-effects and latency differences.

### Variability in Measures of Corticomotor Excitability

As stated above, the after-effects of a-tDCS were characterized by a high degree of inter-individual variability when expressed in terms of changes in MEP amplitude and in cSP duration. In both measures, this variability arose from the presence of a substantial proportion (40–50%) of “non-responders” whose response deviated from to the expected “canonical” response, i.e., they either showed no modulation or a modulation in the opposite direction (e.g., inhibition instead of facilitation). In terms of MEP modulation, our observations on inter-individual variability closely match with those in other recent reports (López-Alonso et al., 2014; Wiethoff et al., 2014), which provides further corroboration that individual responses to a-tDCS are indeed inherently variable when using MEP amplitude as an outcome. As stressed by Wiethoff et al. (2014), this variability has implications for planning experiments or interventions in clinical populations for the expected presence of a substantial proportion of “non-responders” needs to be accounted in designing the intervention. As for changes in the cSP, only a handful of tDCS studies have examined this question and while some reports did find after-effects with tDCS (Hasan et al., 2012; Tremblay et al., 2013), others found no after-effects (Suzuki et al., 2012; Hendy and Kidgell, 2013). In the present report, we did not detect changes in the cSP in our group of participants owing to the presence of large number of non-responders. Our observation on the cSP are consistent with the report of Suzuki et al. (2012) who also failed to detect modulation in cSP after either cathodal or anodal a-tDCS. Thus, the effects of a-tDCS in modulating GABAergic inhibition, as reflected in the cSP duration, remain equivocal given the pattern of inconsistent responses observed within and between studies. To summarize, in agreement with recent reports, a great deal of variability seems to characterize individual responses to a-tDCS, especially when considering variations in MEP amplitude or in cSP duration, leading to marginal after-effects when changes are averaged across all participants.

### Variability in Measures of Transcallosal Inhibition

Contrasting with the large variability seen for MEPs, changes in LTI and DTI were correspondingly far more consistent both in terms of magnitude and direction. This smaller variability allowed for detection of significant after-effects post-tDCS, which were indicative of an increased excitability of the transcallosal pathway in the stimulated hemisphere. To our knowledge, this study is the first to report a lasting modulation in the transcallosal inhibitory drive from the stimulated hemisphere towards the non-stimulated hemisphere, as reflected in the shorter LTI and prolonged DTI observed post-tDCS. In their 2004 report, Lang et al. (2004) also observed an increased transcallosal inhibitory drive in association with anodal stimulation but only from the non-stimulated hemisphere towards the stimulated hemisphere. To account for this observation, the authors speculated on the possibility of a remote influence of t-DCS on the inhibitory interneurons of the opposite hemisphere receiving transcallosal excitatory projections from the stimulated hemisphere. In the present study, both DTI and LTI changes suggest a more direct action of a-tDCS in increasing the excitability of transcallosal projections from the stimulated hemisphere. The reason as to why Lang et al. (2004) failed to detect a direct effect on the transcallosal pathway of the stimulated hemisphere might be linked with the fact that their tDCS intervention was only applied for 10 min at 1 mA. In this study, we used twice the time and intensity, which may explain the difference given that a higher intensity of tDCS may be required to modulate transcallosal excitability as interhemispheric connections have higher thresholds than corticospinal neurons (Wassermann et al., 1991). Another reason for the discrepancy might be related to methodological differences in eliciting the iSP. Lang et al. (2004) used very high TMS intensity (150% rMT) during mild bilateral contraction of the FDI (50% max), whereas we used the procedure advocated by Giovannelli et al. (2009), see “Materials and Methods” Section, which involves mild contraction of the contralateral hand with maximal contraction of the ipsilateral hand. In spite of these differences, both our observations and those of Lang et al. (2004) converge to indicate that tDCS can potentially induce lasting modulation in the excitability of the transcallosal pathway between motor cortices. One important caveat, however, is the fact that in the absence of a sham condition, we cannot exclude the possibility that other factors (e.g., changes in alertness, tiredness) might have contributed to the observed modulation in transcallosal inhibition.

### Predicting Modulation in Corticomotor Excitability and in Transcallosal Inhibition

Consistent with the work of Hamada et al. (2013) and those of Wiethoff et al. (2014), we found that latency differences derived from TMS proved to be good predictors of individual responses to a neuromodulatory intervention. These predictions concerned changes in MEP amplitude, in particular, and not those affecting the cSP duration, which is somewhat expected given that the two measures reflect different underlying mechanism at the cortical level (i.e., modulation of glutaminergic excitatory transmission vs. modulation by GABA B receptors; Ziemann et al., 2015). Contrary to Wiethoff et al. (2014) we did not find an association between smaller baseline MEP amplitude and larger facilitation post-tDCS, although the authors emphasized that the association was only “borderline”. Thus, the potential role of baseline amplitude in predicting MEP modulation in response to a-tDCS remains equivocal. On the other hand, our observations on the role of latency differences in predicting MEP modulation after a-tDCS resonate strongly with those of Wiethoff et al. (2014), since in both their study and ours, AP-LM latency differences proved to be the best predictor (i.e., compared to the other latency differences) of whether a given individual would show facilitation or inhibition in response to stimulation. In fact, in close correspondence with their results (Wiethoff et al., 2014), our analysis revealed that AP-LM latency differences <2.5 ms were highly predictive of the probability of exhibiting the canonical response to anodal stimulation. Such finding provides corroborating evidence that individual susceptibility to recruit early I-waves, as reflected in small AP-LM differences, is indeed a good predictor of whom is likely to respond favorably to a-tDCS. As discussed by Wiethoff et al. (2014) and others (for a review, see Stagg and Nitsche, 2011), the fact that tDCS is thought to exert its influence on the cell body of pyramidal neurons, where early I-waves are likely generated, might account for the link between AP-LM latency differences and the probability of showing either inhibition or facilitation after a-tDCS. The fact that PA-LM latency differences were also predictive of MEP modulation, though to a lesser degree than AP-LM differences, further point to the interplay between early I-waves and a-tDCS after-effects. As pointed out by Hamada et al. (2013), small differences in PA-LM latency (i.e., 1–2 ms) is suggestive of individuals in whom D-wave or early I-waves can be easily recruited even when the stimulation is delivered in the conventional PA orientation. Consistent with this interpretation, only participants with PA-LM latency differences <2 ms exhibited MEP facilitation (see Figure 3B).

With regards to changes in transcallosal inhibition, although a trend was seen for an inverse relationship between AP-LM differences and DTI changes observed post-tDCS, more observations will be needed to confirm the nature of this relationship, i.e., whether a higher susceptibility to recruit D-waves in response to TMS also reflects a higher probability to show modulation in the transcallosal pathway. There is neurophysiological evidence that corticospinal and transcallosal pyramidal neurons share common neuronal circuitry at the intra-cortical level (Trompetto et al., 2004; Avanzino et al., 2007), and thus, it is possible that individuals who are more likely to express corticospinal facilitation in response to a-tDCS, i.e., owing to easy recruitment of D-wave as evidenced by small AP-ML differences, may also exhibit parallel changes in the transcallosal pathway arising from the stimulated hemisphere. Although still highly speculative this possibility certainly deserves more attention in future studies.

In conclusion, the present study adds further observations with regards to the importance of considering inter-subject variability when planning experiments based on neuromodulatory protocols design to induce lasting changes in corticomotor excitability, such as a-tDCS. Such consideration appears particularly important for tDCS studies when the aim is to modulate motor excitability to enhance motor responses such as in patients with stroke or with Parkinson’s disease. Our report also adds further evidence to corroborate the value of latency differences, as surrogate measures of early and late I-waves recruitment, to predict individual changes in MEP modulation in response to a-tDCS. Finally, our report provides new observations suggesting that a-tDCS can potentially exert modulatory influence on the excitability of the transcallosal pathway originating from the stimulated hemisphere.

## Author Contributions

TWD participated in the design of the study, carried out the data collection, analyzed the data, and drafted the earlier version of the manuscript. MB participated in the design of the study. FT conceived the study, aided with data collection and in drafting and editing the final version of the manuscript. All authors read and approved the final manuscript.

## Conflict of Interest Statement

The authors declare that the research was conducted in the absence of any commercial or financial relationships that could be construed as a potential conflict of interest.

## Footnotes

^1^http://clinicalresearcher.org/software.htm

^2^Investigation Testing Authorization No. 213954, Health Canada.

^3^www.graphpad.com
